# Analysis of socioeconomic gradients in the development of children aged 0–3 years in Fortaleza, Northeastern Brazil

**DOI:** 10.11606/S1518-8787.2018052000525

**Published:** 2018-10-11

**Authors:** Florencia Lopez Boo, Mayaris Cubides Mateus, Suzanne Duryea

**Affiliations:** I Inter-American Development Bank . Washington , DC , EUA

**Keywords:** Child Development, Parenting, Anthropometry, Risk Factors, Socioeconomic Factors, Health Inequalities, Desarrollo Infantil, Antropometría, Conducta Materna, Factores de Riesgo, Factores Socioeconómicos, Desigualdades en la Salud

## Abstract

**OBJECTIVE:**

To identify the socioeconomic gradients in the measures of development and well-being of children under three years of age in Fortaleza, Northeastern Brazil.

**METHODS:**

We compiled information using a socioeconomic survey instrument, collecting anthropometric measurements, observing the home environment, and applying the Denver Test II to 2,755 children aged between zero and 28 months who are potential beneficiaries of the *Cresça com Seu Filho* program in Fortaleza. These children were randomly selected from a universe identified from the administrative record of the *Cadastro Único* of the Ministry of Social Development of Brazil. For the analysis, we reported descriptive statistics, Pearson correlations, and mean differences.

**RESULTS:**

Rates of chronic malnutrition and overweight were 7.0%. The results of the Denver II test indicated that personal social (23%) and language (20%) are the domains in which children have the highest developmental delay, when compared with the international reference sample. Parental practices measured by two sub-scales of the Home Observation of the Environment Inventory were poor, with only 14.0% of families having two or more books in the home and 35.0% of the households reporting having spanked their child in the past three days.

**CONCLUSIONS:**

We identified clear socioeconomic gradients in the anthropometric indicators, parenting practices, and the Denver Test II (especially in the language domain). Children from poorer households, as well as children of mothers with lower education levels, perform poorly on most measures.

## RESUMEN

**OBJETIVO:**

Identificar los gradientes socioeconómicos en medidas del desarrollo y bienestar en menores de tres años en Fortaleza, Nordeste de Brasil.

**MÉTODOS:**

Se recolectó información a través de una encuesta socioeconómica, toma de medidas antropométricas, observación del ambiente en el hogar y aplicación del Test de Denver II de 2.755 niños de cero a 28 meses potenciales beneficiarios del programa *Cresça com Seu Filho* en Fortaleza. Estos niños fueron seleccionados aleatoriamente de un universo identificado a partir del registro administrativo del Catastro Único del Ministerio de Desarrollo Social de Brasil. Para el análisis se reportan estadísticas descriptivas, correlaciones de Pearson y diferencias de medias.

**RESULTADOS:**

Las tasas de desnutrición crónica y el sobrepeso fueron iguales a 7,0%. Los resultados del Test de Denver II indicaron que las áreas en las que los niños presentan un mayor rezago en su desarrollo, cuando se compararon con la muestra de referencia internacional fueron personal-social (23,0%) y lenguaje (20,0%). Las prácticas parentales medidas por dos sub-escalas del *Hom* *e Observation of the* *Enviroment* fueron pobres, solo el 14,0% de las familias reportaron tener dos o más libros en el hogar y 35,0% de los hogares reportaron haberle pegado a su hijo en los últimos tres días.

**CONCLUSIONES:**

Se identifican gradientes socioeconómicos claros en los indicadores antropométricos, las pautas de crianza y en la prueba Denver II (especialmente en el dominio de lenguaje). Los niños pertenecientes a los hogares más pobres, así como hijos de madres con menor nivel educativo, presentan un desempeño bajo en la mayoría de las medidas.

## INTRODUCTION

Child development is determined by factors at the individual, family, and societal level ^1^ ^,^ ^2^ . Within the societal level, we can find initiatives promoted by the government, such as early childhood programs and strategies. According to scientific evidence, they show short-term and long-term benefits ^3^ .

Brazil is one of the most successful countries in the Latin America and the Caribbean (LAC) in reducing the infant mortality rate for children aged from zero to five years. The rate declined from 61 deaths per 1,000 live births in 1990 to 16 in 2015 ^4^ – which represents a decrease twice the amount as other LAC countries. However, efforts must still be made to reduce disparities, since 1,000 of the 5,500 Brazilian cities have a mortality rate below five per 1,000 live births, while 32 cities have a rate above 80 ^5^ .

One of the potential reasons behind this decline in infant mortality is the universal health coverage achieved by Brazil ^6^ within a unified health system. This system provides primary care at the community level through the Family Health Strategy (FHS) program. Bhalotra et al. ^7^ use the gradual expansion of the FHS to cities to show that the restructuring and, in particular, the massive expansion of primary health care since 1995 have contributed to a significant and sustained decline in the infant mortality rate (IMR) and maternal mortality ratio (MMR). Decreases in fetal and neonatal maternal mortality are particularly noteworthy since they were the hardest to be obtained worldwide.

Taking advantage of the FHS framework, the program of home visits *Cresça com Seu Filho* (PCCSF) emerged in Fortaleza, Northeastern Brazil. The program is a pioneer and unique in the country since, in addition to maternal and child health issues, it guides the improvement of child development in a broader sense, with the improvement of stimulation practices in young children provided by mothers and caregivers. The program is inspired by the experience of the *P* *rimeira Infância Mel* *hor* (PIM) program and the theoretical framework of the *International C* *hild Development Pro* *grammes/More Intelli* *gent and Sensitive C* *hild* used in the curriculum developed by the *Universidade F* *ederal de Ceará* . It has the contribution of many features of the Reach Up and Learn model: training, supervision, monitoring, and mentoring of the Community Health Agent (CHA), as well as the “spirit” of the visit, that is, the relation established between the visitor and the caregiver and the child, the way to show activities and to praise, among others ^a^ . The program is focused on mothers with children aged between zero and three years, with low levels of education, residing in low income areas in Fortaleza. Home visits are performed by CHA, who receive intensive training for weekly visits lasting one hour for two years.

There is little evidence in Brazil regarding the state of child development for children under 36 months that go beyond nutrition and health outcomes. The objective of this study was to identify socioeconomic gradients in the measures of development and well-being of children under three years of age in Fortaleza, Northeastern Brazil.

Baseline information was collected from 2,755 children aged between zero and 28 months living in the 18 neighborhoods that make up the Region V of Fortaleza. This information, besides being the starting point for an evaluation, allows us to analyze the determinants of the development both at a physical and a cognitive level.

## METHODS

The research design was a stratified cluster sample of households with children under 28 months and a monthly income of less than R$ 500.01, living in the 18 neighborhoods of Region V of Fortaleza. The primary unit of analysis was the child. The clusters were 630 micro areas, corresponding to the geographical service areas of the PCCSF program. This type of design allows the sampling of children within each group and requires a master list for the sample. The master list was formed by a census of eligible households with support from the information provided in the *Cadastro Único* (CADUNICO), a centralized record covering 40.0% of the population of Brazil used to determine eligibility for social programs. The brief census, carried out between August 1, 2015 and February 3, 2016, can also be used to verify the age of the child and the income of the family. We were able to identify 6,737 children belonging to 6,265 households.

Following power calculations, we determined that a minimum of seven children should be visited between March 29 and July 13, 2016 in each of the 630 micro areas that make up Region V. Of the 6,737 children identified, we made the randomized selection conditional on meeting the inclusion criteria of the program: children aged between zero and 28 months, belonging to families in the CADUNICO, and with low income. The only exclusion criterion was that the children should not present severe disability. We collected the information using a socioeconomic survey and applying instruments that take into account child development and household environment. However, given the mobility of the families and because children were growing out of the target age range, we could only interview 2,755 children, who formed the unit of analysis of this study.

The associated factors were the individual variables of age and sex and the household variables: socioeconomic level ( *per* *ca* *pita* income and education level of mothers), parenting practices (self-reported activities such as reading books, counting numbers, storytelling, singing, and playing), and the quality of the household environment (as measured by two subscales of the Home Observation of the Environment – HOME). We used the anthropometric indicators and the results in the Denver Test II as measures of physical and cognitive development, respectively.

The HOME is an instrument designed to measure the quality of the family environment in the household, from a quantitative and qualitative perspective. It is focused on the child as the main recipient of objects, events, and transactions that occur with family members who surround them ^8^ . The version of HOME for children aged from zero to three years consists of 45 items divided into six subscales. However, to decrease time spent in the household, we only applied the 11 items corresponding to the subscales of receptivity and acceptance (the first six items correspond to the receptivity subscale, while the last five items correspond to the acceptance subscale). In addition, we constructed a score from zero to eleven, in which a higher score indicated that the child was exposed to worse interactions with the parents, who were less sensitive and more punitive. This version corresponded to the one applied in other countries of the region and was constructed and interpreted following the recommendation of Paxson and Schady ^9^ ^,^ ^10^ .

The weight and height measurements of the children were compiled following the internationally accepted recommendations for instruments - scale and height meter - and procedure for the collection of measurements. Based on these measurements and using the official index of the World Health Organization, which establishes the expected patterns according to sex and age, we estimated the following anthropometric indicators: weight for age, height for age, weight for height, and body mass index (BMI). From the indicators, we determined whether the children had low weight [-2 Standard Deviations (SD) in weight for age], chronic malnutrition (-2 SD in height for age), acute malnutrition (-2 SD in weight for height), and overweight (+2 SD in BMI), which may be associated with poor growth and malnutrition.

The Denver Developmental Screening Test II ^11^ is an instrument that, from the performance in 125 tasks or items administered according to age, allows the evaluation of the development of children aged from zero to six years in four areas of function: personal-social (such as getting along with other persons and caring for personal needs), fine motor- adaptive (such as hand-eye coordination, manipulation of small objects, and problem solving), language (such as hearing, understanding, and using language), and gross motor (such as sitting, walking, jumping, and overall large muscle movement).

According to the interpretations suggested in the training manual, Denver II categorizes children in each area as “normal” (no delay - successful performance of items that 90% of children their age can perform - and one maximum warning – no performance of an item that 75% of children their age can perform), suspected of being “delayed” (two or more warnings or one or more delays), or “unstable” (refusal to perform at least one item that is completely to the left of the age line), according to their performance compared to the reference population of the Denver Test II (2,096 US children). As it is a screening test, there is no instruction for assigning scores to the children in the Denver II manual. However, for the purposes of the analyses of this article and following other studies ^12^ ^,^ ^13^ , we assumed that the child can successfully carry out all the items that precede the minimum level and we calculated a score equal to the sum of successful items. This score allows us to make comparisons in time (before and after interventions).

We presented the descriptive statistics of the study variables. We performed an analysis of Pearson’s correlations and we presented mean differences according to the variables of the socioeconomic level.

## RESULTS

Approximately 51% of the children in the sample were males, mean age was 18.7 months, and 10.0% were born preterm. Mothers on average completed 9.4 years of education, the average *per* *capita* income was R$254.16 per month, and 80.0% of the households were beneficiaries of the *Bolsa Família* Program. The most frequent activities to stimulate children were playing (93.0%) and praising (91.0%). In contrast, the least frequent activities were storytelling (34.0%), reading books (36.0%), and counting numbers (54.0%). Approximately 77.0% of the caregivers had yelled at the child and 35% had spanked them. The mean HOME score was 1.8, which indicates that caregivers presented undesirable behaviors in 1.8 of the 11 practices evaluated ( Table 1 ).

**Table 1:** Descriptive statistics of the study sample. Fortaleza, Brazil, 2016.

Variable	M	SD	P10	P90
Sex (1 = male, 0 = female)	0.50	0.50	0	1
Age (months)	18.70	5.90	11	26
Preterm birth	0.10	0.30	0	0
Years of education of the mother	9.40	2.80	5	12
*Per* *capita* household income	254.10	202.50	16.97	518.17
Household is part of the *Bolsa Família* Program	0.80	0.40	0	1

In the last 3 days, an adult				

Read books	0.40	0.50	0	1
Told stories	0.30	0.50	0	1
Sang	0.80	0.40	0	1
Took the child outside the house	0.90	0.30	0	1
Played	0.90	0.20	1	1
Counted numbers	0.50	0.50	0	1
Yelled	0.80	0.40	0	1
Spanked	0.30	0.50	0	1
Praised	0.90	0.30	1	1

HOME score	1.80	1.80	0	4

Anthropometric indicators				

Weight for age (standardized)	0.30	1.30	-1.30	1.93
Height for age (standardized)	-0.00	1.30	-1.67	1.67
Weight for height (standardized)	0.40	1.50	-1.53	2.34
Body mass index (standardized)	0.40	1.60	-1.60	2.42
Low weight	0.00	0.20	0	0
Chronic malnutrition	0.10	0.20	0	0
Acute malnutrition	0.10	0.20	0	0
Overweight	0.10	0.20	0	0

Denver II Test – Delay rate				

Personal-social	0.23	0.40	0	1
Fine motor	0.10	0.30	0	1
Language	0.20	0.40	0	1
Gross motor	0.10	0.30	0	1

HOME: Home Observation of the Environment

On average, the anthropometric indicators showed a performance above expectations, except for height for age (whose average was negative). We found similar rates of 7.0% for chronic malnutrition and overweight. On the other hand, the results of the Denver Test II indicated that, when compared with the international reference sample, the children presented the highest delays in the developmental areas of personal-social (23.0%) and language (20.0%).

Table 2 shows the correlations between the individual and household variables with the standardized score for each of the areas of function and the Denver Test II. Regarding the correlation between the Denver II scores and the socioeconomic variables, we observed that higher number of years of education of the mother and higher *per* *capita* income were positively associated with all areas of function, except for personal-social, which, although positively associated with higher *per* *capita* income, was not significant. Similarly, coefficients were higher for income than for education of the mother. Being a beneficiary of the *Bo* *lsa Família* Program was also associated with a low language score. Although chronic malnutrition was negatively associated with all areas of function, the coefficient was only statistically significant for gross motor. A possible explanation comes from the point of view of the required energy (low in malnourished children) for the movements that the test demands for this domain. Low weight was negatively and significantly associated with fine motor and language, while overweight was associated with personal-social and fine motor ( Table 2 ).

**Table 2:** Pearson correlations between Denver II and individual and household variables. Fortaleza, Brazil, 2016.

Variable	Low weight	Chronic malnutrition	Acute malnutrition	Overweight	Personal-social	Fine motor	Language	Gross motor
Personal-social	-0.02	-0.02	-0.03	-0.04 ^b^	1	0.32 ^a^	0.37 ^a^	0.33 ^a^
Fine motor	-0.04 ^b^	-0.01	-0.04 ^b^	-0.04 ^b^	0.32 ^a^	1	0.3 ^a^	0.37 ^a^
Language	-0.05 ^a^	-0.01	-0.02	-0.02	0.37 ^a^	0.3 ^a^	1	0.31 ^a^
Gross motor	-0.03	-0.04 ^b^	-0.02	-0.02	0.33 ^a^	0.37 ^a^	0.31 ^a^	1
Sex	0.05 ^a^	0.03 ^c^	0.02	0.04 ^c^	-0.18 ^a^	-0.03	-0.1 ^a^	0.06 ^a^
Age	-0.01	-0.01	0.03	0.01	0	0	0	0
Preterm birth	0.07 ^a^	0.06 ^a^	0.05 ^b^	0.04 ^b^	-0.05 ^a^	-0.07 ^a^	-0.09 ^a^	-0.09 ^a^
Education of the mother	-0.04 ^b^	-0.07 ^a^	-0.08 ^a^	-0.07 ^a^	0.04 ^c^	0.05 ^b^	0.08 ^a^	0.06 ^a^
*Per* *capita* income	-0.03 ^c^	-0.01	-0.03	-0.03 ^c^	0.03	0.04 ^b^	0.05 ^b^	0.03 ^c^
Beneficiary of *Bolsa Família* Program	0.02	0.03	0.01	0.01	0.02	0	-0.03 ^c^	-0.01
HOME	0.01	0.01	-0.01	0.01	-0.17 ^a^	-0.13 ^a^	-0.17 ^a^	-0.13 ^a^
Read books	-0.02	0	-0.03	-0.02	0.1 ^a^	0.05 ^a^	0.08 ^a^	0.02
Told stories	-0.01	0	-0.01	0	0.1 ^a^	0.05 ^a^	0.09 ^a^	0.02
Sang	-0.06 ^a^	0	-0.07 ^a^	-0.07 ^a^	0.03	0.02	0.09 ^a^	0.05 ^a^
Took the child outside the house	-0.05 ^b^	-0.05 ^a^	-0.01	-0.02	0.05 ^b^	0.04 ^b^	0.04 ^b^	0.03
Played	-0.02	-0.03 ^c^	0.01	0.01	0.03 ^c^	0.02	0.02	0.04 ^b^
Counted numbers	-0.04 ^b^	-0.04 ^b^	-0.05 ^b^	-0.04 ^b^	0.07 ^a^	0.07 ^a^	0.08 ^a^	0.06 ^a^
Yelled	-0.04 ^b^	-0.06 ^a^	-0.02	-0.01	0.01	0.04 ^b^	0.02	0.1 ^a^
Spanked	-0.01	0	-0.01	-0.01	0.02	0	0.01	0.03 ^c^
Praised	-0.03 ^c^	0	0	-0.01	0.07 ^a^	0.05 ^b^	0.08 ^a^	0.09 ^a^

HOME: Home Observation of the Environment

^a^ p < 0.01

^b^ p < 0.05

^c^ p < 0.1

Positive parenting practices such as reading books and counting numbers had a positive and significant association most of the time. The HOME score was negatively associated and always significant. HOME is coded in such a way so that low scores in the inventory mean better parenting practices.

When investigating the association between indicators of child physical development, such as low weight, chronic or acute malnutrition, and overweight, with individual and household factors, through Pearson correlations ( Table 2 ), being male and being born preterm had a positive and statistically significant association with all indicators (with the exception of acute malnutrition). On the other hand, the years of education of the mother had a negative and significant association with all indicators. Although *per* *capita* income was negatively associated with all indicators, it was only significant for low weight and overweight. Being part of the *Bolsa Família* Program, although with a positive association, was not statistically significant in any of the cases ( Table 2 ).

**Figure 1:**
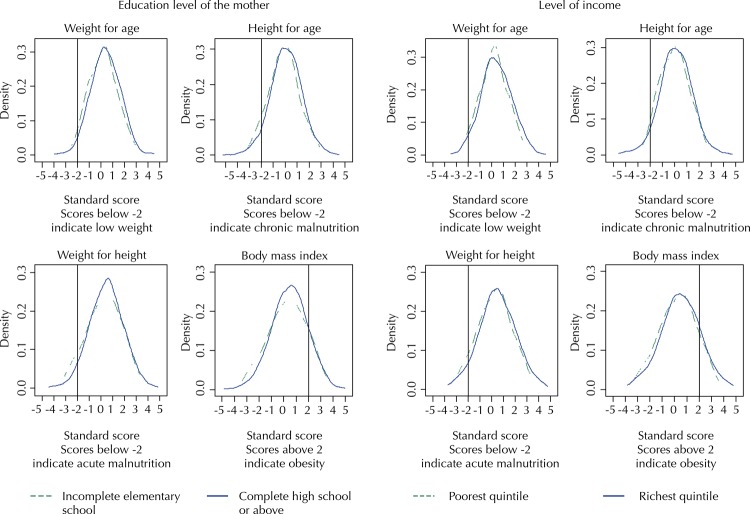
Distribution of anthropometric indicators according to the education level of the mother and the level of household income. Fortaleza, Brazil, 2016.

The distributions of socioeconomic gradients in the physical development for the most vulnerable children (with mothers with incomplete elementary school or belonging to households from the poorest quintile according to *per* *capita* income) were slightly to the left for all anthropometric indicators ( Figure 1 ). However, the difference in means (evaluated using the t-test) was only statistically significant for the indicators of overweight and chronic malnutrition according to the education level of the mother.

**Figure 2:**
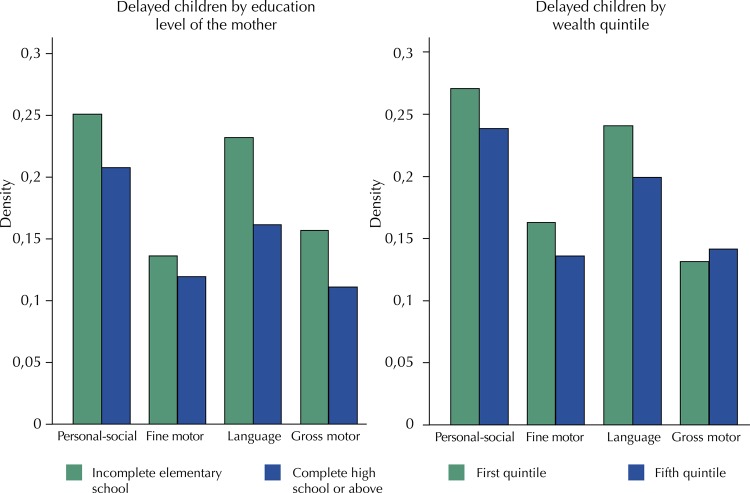
Socioeconomic gradients in the percentage of children suspected of being “delayed”. Fortaleza, Brazil, 2016.

For all domains, a higher percentage of children from mothers whose education level was lower than completed high school were suspected to be “delayed”. In the same sense, in the personal-social, fine motor, and language areas, more children belonging to the first income quintile were suspected to be “delayed” ( Figure 2 ).

**Figure 3:**
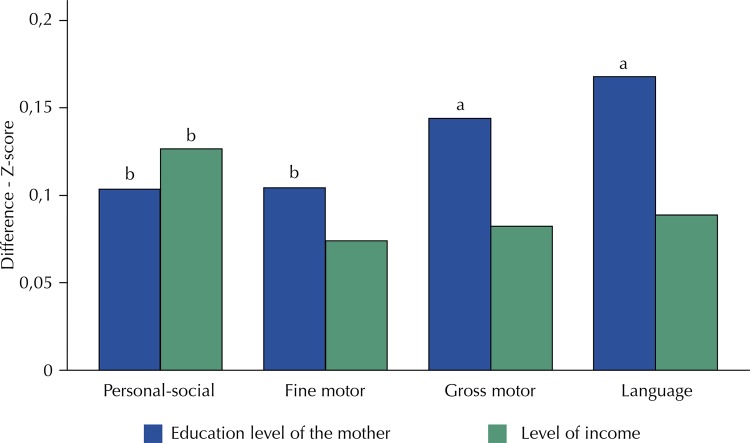
Socioeconomic gradients in the Denver II. Fortaleza, Brazil, 2016.

We presented the results corresponding to the internal standardization of the scores for each of the domain areas to eliminate the age effect from the crude scores. Specifically, the standardization of scores corresponds to a z-score (we subtracted the mean of the scores and divided by the standard deviation for each month of the age of the sample children). There were significant differences in all dimensions between children whose mothers had an education level equal to or higher than complete high school and children whose mothers had incomplete elementary school. We found the largest differences in the language area (SD = 0.17), followed by gross motor (SD = 0.14), fine motor (SD = 0.10), and personal-social (SD = 0.10). We also found differences for all areas in relation to the level of income, but they were only significant for the personal-social area (SD = 0.13) ( Figure 3 ).

Education level of the mother: complete high school or above versus incomplete elementary school.

Level of earnings: richest quintile versus poorest quintile.

^p^ < 0,01

^b^ p < 0,05

Finally, Table 3 presents the results by socioeconomic level of the HOME score. We observed that there was a difference of 0.33 points (with a significance level of 1%) between the children of the poorest quintile and the children of the richest quintile. This difference was greater among girls, who, in relative terms, had a better home environment than boys for all wealth quintiles, except for the third quintile. For the education level of the mother, there was a difference of 0.75 points (SD = 0.30) in the scale between the children whose mothers had completed high school or above and the children whose mothers had incomplete elementary school or less. Again, the difference was greater among girls who, in relative terms, were exposed to better stimulation than boys for the two highest education levels.

**Table 3:** Socioeconomic gradients in the HOME. Fortaleza, Brazil, 2016.

Variables	Total	Men	Women
By level of Income			
First quintile (poorest, Q1)	1.98	1.98	1.97
Second quintile	1.87	2.00	1.74
Third quintile	1.91	1.90	1.93
Fourth quintile	1.61	1.72	1.50
Fifth quintile (richest, Q5)	1.65	1.70	1.60
Test Q1 = Q5 (p-value)	0.00	0.07	0.02

By education level of the mother			

Incomplete elementary school (E1)	2.31	2.26	2.36
Complete elementary school or incomplete high school	2.01	2.02	1.99
Complete high school or above (E3)	1.56	1.64	1.48
Test E1 = E3 (p-value)	0.00	0.01	0.00

HOME: Home Observation of the Environment

## DISCUSSION

The LAC region is undergoing a paradigm shift. Governments believe more and more that healthy parenting practices at an early age are not just a private matter, but one of concern for public policies. As a consequence, governments, including the Brazilian government, look for ways not only to improve parenting practices (with programs such as the *Programa* *Cresça com Seu Filh* *o,* whose population was studied in this article) but also to generate diagnoses with accurate data on factors associated with the development of very young children.

We found that most families are part of the *Bolsa Família* program, which indicates good targeting of the PCCSF, and 10.0% of the children were born preterm (a somehow higher level than the official rate). Parenting practices are poor, with only 14.0% of families reporting having two or more books in their homes and 35.0% of households reporting having spanked their child in the last three days. We found clear socioeconomic gradients in the anthropometric indicators, parenting patterns, and the Denver Test II, which is in line with other LAC countries ^1^ . We found the largest differences in the language area. In addition, there is a significant difference between the children of the poorest quintile and the children of the richest quintile in the HOME. This difference is greater among girls, who are exposed to better stimulation than boys for the two highest education levels.

The children in Fortaleza are exposed to better family environments than in Ecuador, Peru, Nicaragua, and the Caribbean throughout the distribution of income and education of the mother ^14^ . We highlight that this difference is greater for girls. This indicates that girls are exposed to better stimulation than boys in the higher education levels.

Habits such as early reading and storytelling generate benefits in children, such as better performance in early school years and establishment of closer relationships with their primary caregivers ^4^ . When comparing these figures with other studies in the region, we found that Fortaleza is only below Guyana, Peru (rural), and Nicaragua (rural), while other countries such as Jamaica and Santa Lucia reach 88.0%. On the other hand, disciplinary strategies such as severe punishment (such as hitting children with objects, striking a child with a fist, or striking a child in the face or chest) can cause pshysical and psychological damage. For the region, the percentage of parents who use severe punishment is between 40.0% and 50.0%. Thus, although we cannot identify the type of punishment (mild or severe) in our sample, the result found is within the range for the LAC region.

On the other hand, when comparing the results of the socioeconomic gradients in the development of children with the results presented in Berlinski and Schady ^4^ , we could see that the socioeconomic levels in this Fortaleza sample are smaller in magnitude than those found in other countries. However, our sample is clearly pro-poor and thus the gradients will be smaller than in representative samples of the entire spectrum of the distribution of income. In this sense, Lopez Boo et al. ^b^ , using the same version of Denver II in another study, have found significant differences by wealth quartiles and education level of the mother, with magnitudes similar to those of Fortaleza. The exception is language, in which the gradient by the education level of the mother is twice that found in Fortaleza.

This study has limitations. The analysis was performed in a specific context of low quality of family environment because of the focus of the PCCSF. Although we were able to explore the variation of the scores within this low quality range in our correlation study, it is possible that the low general variability in the quality of the environment “masks” some of the low correlations in our analysis. Therefore, we recommend the replication of this study in other contexts to ensure the external validity of the results obtained here. Similarly, the study only measures four dimensions of child development, and more and more research studies propose measuring executive functions. However, this type of measurements for young children is a great challenge because of the lack of consensus indicators in the literature. In contrast, the strength of the study is the inclusion of the measurement of child development outcomes using the Denver Test II. Similarly, this measurement was made together with a large number of socioeconomic and demographic variables. In addition, it is innovative as it includes the HOME measure, which has not been applied at a wide scale in the LAC region.

Our findings are useful in informing policy decisions about the need for early childhood programs that can be taken to scale. Currently, there are programs that try to improve parenting practices, but they do not take into account the large deficits in language, or the differential parenting practices according to the sex of the child. Additionally, the use of indicators such as HOME can allow the detection of critical aspects of the quality of the home environment and guide the design of training programs for visitors of this type of programs.

## Footnotes

a Lopez Boo F, Encina J. Informe de capacitaciones de *Reach Up* en Fortaleza. Working Paper. 2015. Unpublished data. To obtain them, contact the corresponding author.

b Lopez Boo F, Bando R, Leer J, Lee X. Características de los niños nicaragüenses menores de seis años, sus cuidadoras, su hogar y su comunidad: línea de base del PAIPPI. 2013. Unpublished data. To obtain them, contact the corresponding author.
